# Matrix metalloproteinase-2 is elevated in midtrimester amniotic fluid prior to the development of preeclampsia

**DOI:** 10.1186/1477-7827-7-85

**Published:** 2009-08-23

**Authors:** Michal Lavee, Shlomit Goldman, Etty Daniel-Spiegel, Eliezer Shalev

**Affiliations:** 1 Laboratory for Research in Reproductive Sciences, Department of Obstetrics and Gynecology, Ha'Emek Medical Center, 18101, Afula, Israel; 2 Rappaport Faculty of Medicine, Technion-Israel Institute of Technology, Haifa, Israel

## Abstract

**Objective:**

To evaluate levels of matrix metalloproteinases (MMP) and their inhibitors (TIMP) in second trimester amniotic fluid of women with hypertensive disorders compared to normotensive women.

**Study Design:**

Amniotic fluid was obtained from 133 women undergoing genetic second trimester amniocentesis. Zymography was performed for MMP characterization and an MMP-2 ELISA kit was used to determine MMP-2 levels. TIMP-2 expression was evaluated using western blot.

**Results:**

Mean amniotic fluid MMP-2 and TIMP-2 levels were significantly higher in women who developed a hypertensive disorder compared to normotensive women (P < 0.0004 and P < 0.01, respectively). When subdivided into subgroups, amniotic fluid from women who eventually developed preeclampsia or superimposed preeclampsia showed significantly higher MMP-2 levels than normotensive women (P < 0.05). However, no statistical difference in MMP-2 levels was found between patients with gestational hypertension and normotensive patients.

**Conclusion:**

Higher amniotic fluid MMP-2 and TIMP-2 levels are found in women who eventually develop preeclampsia.

## Background

Preeclampsia is a multi-system disorder of pregnancy characterized by hypertension, proteinuria and generalized systemic vasoconstriction. The disorder is diagnosed in the latter half of pregnancy, effects about 5% of pregnancies and accounts for considerable mortality and morbidity [1].

Several models have been proposed for the pathogenesis of preeclampsia. [2-4]. Prevailing evidence suggest insufficient trophoblast invasion of the maternal spiral arteries as the main pathogenesis of the disease. Whereas in normal pregnancy the luminal diameter of the spiral arteries is greatly increased and the vascular smooth muscle is replaced by trophoblast cells, in preeclamptic pregnancies this process is deficient. The resulting under-perfused placenta attempts to compensate by promoting the secretion of factors into the maternal circulation causing systemic alterations in endothelial cell function, accounting for the clinical syndrome of preeclampsia. Although this hypothesis is widely accepted, the molecular mechanisms that regulate this pathological process are still controversial.

The role of matrix metalloproteinases (MMPs) in the pathogenesis of preeclampsia has been given much attention in recent years [5-9]. These extra-cellular matrix-remodeling enzymes, balanced by their tissue inhibitors (TIMP), are essential effectors of developmental processes including cell migration, cell proliferation, apoptosis and tissue morphogenesis [10]. MMPs play an important role in endothelial cell invasion, angiogenesis and in tumor progression [11-14].

A reduction in the content and activity of MMPs in the umbilical cord artery was found in preeclamptic pregnancies compared with normotensive pregnancies [7]. Higher levels of MMP-2 have been demonstrated in plasma of women with preeclampsia [8]. More recent studies have shown altered serum MMP-2 levels prior to the onset of preeclampsia. [15].

In an effort to study the MMP profile before the onset of clinical preeclampsia, and in a more reliably fetal-placental environment, we designed our study to measure MMP in second trimester amniotic fluid of women with normotensive pregnancies and those who eventually develop preeclampsia.

## Methods

### Patient selection

Following approval by the local ethics committee, and informed consent from each patient, 290 samples of amniotic fluid were collected from singleton pregnancies during second trimester genetic amniocentesis between June 2005 and January 2006. Ha'emek Medical Center is the largest medical center in the area and provides genetic amniocentesis for most women living in this part of Israel. However, several smaller hospitals in the area provide obstetric services. For this reason, approximately half of the women who underwent amniocentesis in our facility delivered elsewhere. Consequently, pregnancy follow up was possible in 150 women who chose to deliver at Ha'emek Medical Center. In these cases, data collected included gestational age at delivery, method of delivery, obstetrical complications, fetal gender, weight, apgar score and pH value, when performed. Five of these 150 women were omitted from the study (four elective terminations of pregnancy due to karyotype abnormalities and one late missed abortion). Twelve cases in which spontaneous preterm delivery occurred were excluded from the study as well.

Preeclampsia (PET) was defined as hypertension with a systolic blood pressure >140 mmHg and/or a diastolic blood pressure >90 mmHg in association with proteinuria [24 h urinary protein exceeding 300 mg per 24 h or persistent 30 mg/dl (1+dipstick) in random urine samples] with or without edema. Gestational Hypertension (GH) was defined by hypertension with systolic blood pressure >140 mmHg and/or diastolic blood pressure >90 mmHg, appearing for the first time after midpregnancy, without proteinuria. Superimposed preeclampsia (sPET) was defined as women with chronic hypertension (hypertension prior to pregnancy or early in pregnancy, i.e. <20 weeks' gestation) and new onset proteinuria.

### Sample preparation

Amniotic fluid was collected from 290 women who underwent genetic second trimester amniocentesis. One milliliter of amniotic fluid was removed and centrifuged at 2600 rmp for 10 minutes before storing at -80°C.

### Substrate-gel-electrophoresis (zymography)

Amniotic fluid, molecular mass markers (10 ml), and standard commercial gelatinases A and B (7 ml; Oncogene Science, Cambridge, MA, USA) were diluted with sample buffer (5% SDS, 20% glycerol in 0.4 mol/l Tris, pH 6.8). Samples were electrophoresed through a 10% polyacrylamide gel containing 1 mg/ml gelatin. After electrophoresis, gelatin gels were washed twice in 2.5% Triton X-100. Gelatin gels were incubated for 24 h at 37°C in 0.2 mol/l NaCl, 5 mmol/l CaCl2, 0.02% Brij 35 and 50 mmol/l Tris, pH 7.5). The buffer was decanted and the gel stained with Coomassie Blue for 10 min at room temperature. Areas where proteolytic activity degraded the gelatin are seen as absence of staining. Identification of each gelatinase band was in accordance with its molecular weight and commercial standards (data not shown). These were quantified using the BioImmaging gel Documentation system (Dinco & Renium, Jerusalem, Israel) endowed with TINA software (Raytest, Staubenhardt, Germany).

### Western blot analysis

Amniotic fluid and molecular mass marker (10 ml) were diluted with sample buffer (5% SDS, 20% glycerol in 0.4 mol/l Tris, pH 6.8) and subjected to 10% polyacrylamide gel electrophoresis. After electrophoresis, the gel was transferred onto 0.45 mm nitrocellulose membranes (Scheicher & Schuel, Dassel, Germany). Nonspecific binding sites were blocked by incubating the nitrocellulose membranes overnight with 20% non-fat milk. The membranes were then washed twice with Tris-buffered saline, containing 0.5% Tween-20, and incubated with mouse antihuman TIMP-1 antibody (1.0 mg/ml; Oncogene Science, Cambridge, MA, USA). The membranes were washed with Tris-buffered saline, containing 0.5% Tween-20 and incubated with horseradish peroxidase-conjugated anti-mouse rabbit secondary antibody (Jackson ImmunoResearch, West Grove PA, USA), then detected by enhanced chemiluminescence's (ECL; Amersham International) and quantified by densitometry as above.

### MMP-2 Elisa-kit

Total MMP-2 (pro and active forms) levels were measured using EIA commercial kits (cat. RPN2617, Danyelbiothech, Rehovot, Israel), according to the manufacturer's recommendations. Optical density was measured at 450 nm.

### Statistical analysis

Data are presented as mean ± SEM. Statistical analysis of data was performed using Student's t-test for parametric or Chi square test for non-parametric continuous data when comparing two groups. SPSS statistical software was used. A level of P < 0.05 was considered to be significant.

## Results

Indications for amniocentesis were: maternal age 54%, pathological triple test 27%, abnormal sonographic findings 16% and other indications (prior history or family history of chromosomal abnormality and patient request) 3%. A hypertensive disorder was diagnosed in 17 out of 133 pregnancies (12.8%). Five women developed GH (3.7%), five developed PET (3.7%) and 7 developed sPET (5.3%). In this study group all women diagnosed with chronic hypertension eventually developed superimposed PET. Clinical characteristics and pregnancy outcome of the study groups are summarized in Table 1. In all cases, both in the hypertensive and the normotensive groups, no major fetal malformations were reported and normal karyotype had been documented. No significant difference in maternal age, gestational age at amniocentesis, method of delivery, cord pH or apgar scores was documented between the two groups. The hypertensive group presented significantly lower gestational age at delivery and a lower birthweight compared to the control group (Table 1). Small for gestational age newborns (below 10^th ^percentile) were delivered by 9 of 116 women in the normotensive group(8%), compared with 3 of 17 women in the hypertensive group (18%).

**Table 1:** Clinical characteristics and pregnancy outcome in the hypertensive and normotensive groups.

	Hypertensive (n = 17) Mean ± SD	Normotensive (n = 116) Mean ± SD	P
Maternal age	36.53 ± 4.64	34.04 ± 5.11	0.06
Gest. Age at AC	18.88 ± 3.04	19.38 ± 3.12	0.53
**Gest. Age at delivery**	**36.59 ± 2.79**	**39.54 ± 1.23**	**<0.01**
Method of delivery			
Vaginal delivery	10 (58.8%)	82 (70.7%)	
Cesarean section	7 (41.2%)	34 (29.3%)	0.40
Fetal gender			
Male	7 (41.2%)	63 (54.3%)	
Female	10 (58.8%)	53 (45.7%)	0.44
**Birthweight**	**2862 ± 812**	**3389 ± 436**	**< 0.01**
Apgar score			
1 minute	9.25	9.24	0.96
5 minute	9.94	9.97	0.43
Cord pH	7.34	7.34	0.97

AC: amniocentesis.

### Zymographic characterization of amniotic fluid MMP in hypertensive and normotensive pregnancies

**Figure 1:**
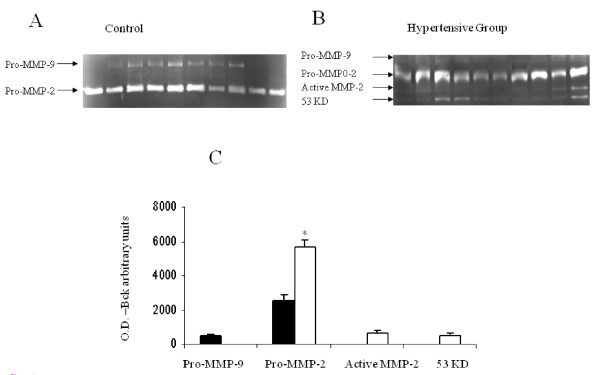
**MMP profile in second trimester amniotic fluid of women with normotensive pregnancies and women who developed a hypertensive disorder in pregnancy**. Representative zymography gels. **A **- Secretion of Pro-MMP-9 and Pro-MMP-2 obtained from second-trimester amniotic fluid of women with normotensive pregnancies. **B **- Secretion of Pro-MMP-9, Pro-MMP-2, Active-MMP-2 and a fourth band (53 kDa) obtained from second-trimester amniotic fluid of women who developed a hypertensive disorder in pregnancy. **C **- Bar graph representing mean ± SEM of MMP secretion, expressed as OD - background, from 37 amniotic fluid samples. Black bars represent normotensive pregnancies (n = 20). White bars represent hypertensive pregnancies (n = 17). T-test, *P < 0.001.

The results are presented in Figure 1. Zymography of amniotic fluid obtained from women with normotensive pregnancies (n = 20) showed the presence of both 92 kDa Pro-MMP-9 (479 ± 79.3 optical density/background) and 72 kDa Pro-MMP-2. A different MMP profile was observed in the hypertensive group (n = 17). In this group, ProMMP-9 was not demonstrated, while Pro-MMP-2 was significantly higher (2556 ± 228 versus 5686 ± 413 optical density/background, respectively; P < 0.001). In addition, a clear band of active MMP-2 (689 ± 101 optical density/background) and a fourth band (53 kDa), were observed in amniotic fluid collected from women in the hypertensive group. This forth band (507 ± 105 optical density/background) could possibly represent MMP-3 (Stromelisyn-1).

### Amniotic fluid MMP-2 levels in hypertensive and normotensive pregnacies

The hypertensive group included all 17 women who developed hypertensive disorder during pregnancy. For the control group we chose 47 random samples from the 116 amniotic fluid samples obtained from normotensive women. Clinical characteristics and pregnancy outcome of this normotensive subgroup did not differ significantly from the entire normotensive group.

**Figure 2:**
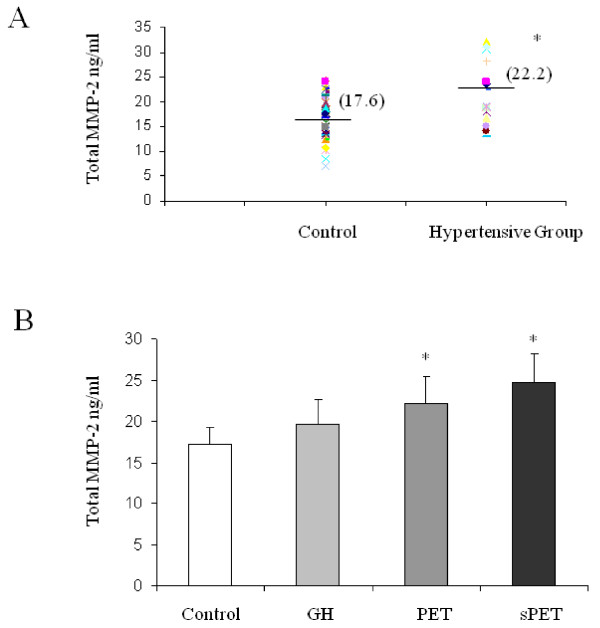
**Quantitative MMP-2 secretion in second trimester amniotic fluid of women with normotensive pregnancies and women who developed a hypertensive disorder in pregnancy**. MMP-2 secretion expressed as ng/ml MMP-2. **A **- MMP-2 secretion, as detected by ELISA kit (detected both pro and active forms) of amniotic fluid samples from women with normotensive pregnancies (n = 47) and women who developed a hypertensive disorder in pregnancy (n = 17). T-test, *P < 0.0004. **B **- Bar graph representing mean ± SEM quantitative MMP-2 secretion level divided into subgroups. T-test, *P < 0.05.

Mean amniotic fluid MMP-2 levels in the hypertensive group and the control group are presented in figure 2A. Mean amniotic fluid MMP-2 levels were significantly higher in women who developed a hypertensive disorder in pregnancy compared with normotensive pregnancies (22.3 ± 1.3 ng/ml versus 17.2 ± 0.6 ng/ml, respectively; P < 0.0004). We further subdivided the hypertensive group into GH, PET, and sPET. The results are presented in figure 2B. In amniotic fluid of women who eventually developed PET or sPET, MMP-2 levels were significantly higher compared to women with normotensive pregnancies (22.2 ± 1.2 ng/ml and 24.8 ± 1.3 versus 17.2 ± 0.6 ng/ml, respectively; P < 0.05). Amniotic fluid MMP-2 levels were, however, not significantly higher in the GH group compared to the control group (figure 2).

### Amniotic fluid TIMP-2 levels in hypertensive and normotensive pregnancies

**Figure 3:**
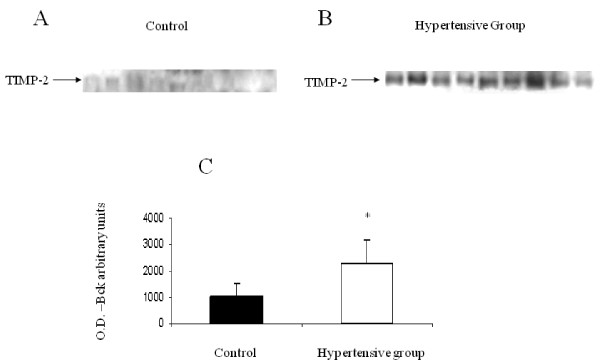
**TIMP-2 expression in second trimester amniotic fluid of women with normotensive pregnancies and women who developed a hypertensive disorder in pregnancy**. Representative Western blot. **A **- Expression of TIMP-2 in second-trimester amniotic fluid obtained from women with normotensive pregnancies. **B **- Expression of TIMP-2 in second-trimester amniotic fluid obtained from women who developed a hypertensive disorder in pregnancy. **C **- Bar graph representing mean ± SEM of TIMP-2 expression from 37 amniotic fluid samples. Black bar represents normotensive pregnancies (n = 20). White bar represents hypertensive pregnancies (n = 17). T-test, *P < 0.001.

The results, presented in Figure 3, showed that TIMP-2 was detected in amniotic fluid of both groups of women (20 normotensive and 17 hypertensive). The level of TIMP-2, however, was significantly higher in women who developed a hypertensive disorder, compared with normotensive women (2277 ± 135 versus 1043 ± 92 optical density, respectively; P < 0.01).

## Conclusion

Preeclampsia, a pregnancy-specific disorder, contributes substantially to perinatal morbidity and mortality of both mother and newborn [1].

This study was designed to test second trimester amniotic fluid MMP levels in women who develop preeclampsia, prior to clinical presentation of the disease. Our decision to study MMP-2 levels in amniotic fluid stemmed from the fact that the amniotic compartment contains MMP-2 which is most reliably fetal in origin, whereas maternal serum MMP-2 could be of placental origin or influenced by non-pregnancy related causes, some yet to be determined

We showed significantly higher levels of MMP-2 in amniotic fluid of women who developed a hypertensive disorder compared to normotensive women. These findings were consistent in both the zymographic presentation and the Elisa test.

Higher levels of MMP-2 were found in the plasma of non-pregnant patients with hypertension [16]. Several other studies have found increased plasma MMP-2 levels in women with preeclampsia [8,15]. Myers at al [15] measured plasma levels of MMP-2, MMP-9 and TIMP-1,2 at three different points of gestation (22 weeks, 26 weeks, at delivery). Women who eventually developed preeclampsia showed increased levels of MMP-2 at 22 weeks but not at 26 weeks. This study supports our results of elevated amniotic fluid MMP-2 during the second trimester, prior to clinical presentation of preeclampsia.

Others found higher total VEGF (vascular endothelial growth factor), MMP-2, and endothelin-1 concentrations in umbilical vein endothelial cells of women with preeclampsia and showed significant positive correlations between them [17].

We found increased levels of TIMP-2 in amniotic fluid of women who eventually developed a hypertensive disorder compared with normotensive women. These results contradict the findings of Myers et al, in which TIMP-2 levels in plasma of women who developed preeclampsia were not quantifiable. This can be explained by the different origin of the tested samples. The mammalian tissue inhibitor of metalloproteinase (TIMP) family consists of four proteins (TIMP-1, 2, 3, and 4), and regulates the activity of MMPs that are capable of degrading extracellular matrix. It has been hypothesized that, together with MMP-2 and MT1-MMP (localized to the cell surface membrane), TIMP-2 is vital for initiating angiogenesis [18]. Since early placentation involves trophoblast invasion into blood vessels and angiogenesis, we might suggest a role for TIMP-2 in abnormal placentation and angiogenesis. Merchant et al [6] found a significantly enhanced release of MMP-2, TIMP-1, and TIMP-2 from umbilical vein endothelial cells of preeclamptic women compared to normotensive women. These findings may provide an explanation as to the origin of the increased amniotic fluid MMP-2 and TIMP-2 we found in our study. Higher TIMP-1 and TIMP-2 levels were also found in the plasma of women with GH compared with normotensive women [19].

When further subdivided into groups of GH, PET and sPET, we found a significant difference in MMP-2 amniotic fluid levels between normotensive women and women who developed PET or sPET. No significant difference, however, was found between normotensive women and women with gestational hypertension. Preeclampsia is a systemic disease characterized not only by hypertension but also by increased vascular resistance, diffuse endothelial dysfunction, proteinuria, and coagulopathy. In the absence of severe disease manifestations, discrimination between PET and GH may be difficult. This distinction is often made solely on the basis of urine protein determination, frequently by dipstick protein measurement, which is recognized to be an imperfect surrogate for 24-hour measurements. Preeclampsia and gestational hypertension may represent different manifestations of one disease process and some consider gestational hypertension to be a precursor to preeclampsia [20]. The theory that hypertension in pregnancy may represent a spectrum of manifestations, ranging from mild (GH) to severe symptoms (PET, sPET), may explain our results showing progressive increase in MMP-2 levels between the GH group and PET, sPET groups.

Whether this could contribute to the increased amniotic fluid MMP-2 level in women with chronic hypertention and superimposed PET in unclear. In our study, all women with chronic hypertension developed superimposed PET. Therefore, we were not able to measure amniotic fluid levels of women with chronic hypertension without sPET.

Our study group consisted of women undergoing genetic second trimester amniocentesis, mostly due to maternal age (mean age 34 yrs). This fact may explain the relatively high prevalence of hypertensive disorders (12% in our study, compared with 5% in the literature), as well as the relatively high percentage of chronic hypertension with superimposed preeclampsia (4.8%). We acknowledge that this is a high-risk population and is not comparable to the general population. We were not able to study MMP-2 levels in women with chronic hypertension without superimposed preeclampsia. This would be an interesting subgroup of women to include in further studies.

Although statistical significance has been achieved, one of the limitations of our study is the small number of hypertensive women. The subdivision into subgroups (GH, PET, sPET) was further limited by smaller numbers. This is a preliminary study which should be expanded in order to validate the results.

Another limitation of the study pertains to the invasiveness of the amniocentesis procedure. The invasive nature of the procedure precludes the use of amniotic fluid MMP-2 levels as a screening test for preeclampsia, but rather as a means of gaining further insight into the pathophysiology of the disease.

In conclusion, higher levels of MMP-2 and TIMP-2 are found in the amniotic fluid of women prior to presentation of preeclampsia. The possibility that these proteins may have a role in the development of preeclampsia needs a further study.

## Competing interests

The authors declare that they have no competing interests.

## Authors' contributions

ML conceived of the study, participated in its design, carried out the laboratory analyses and drafted the manuscript. SG participated in the design, coordinated the laboratory analyses and helped to draft the manuscript. EDS participated in the design, helped to recruit patients and to draft the manuscript. ES participated in conceiving and in the design, coordinated the study and edited the manuscript. All authors read and approved the final manuscript.
